# Gene-specific differential response to anti-apoptotic therapies in zebrafish models of ocular coloboma

**Published:** 2011-06-04

**Authors:** Cheryl Y. Gregory-Evans, Mariya Moosajee, Xianghong Shan, Kevin Gregory-Evans

**Affiliations:** 1 Department of Ophthalmology and Visual Sciences, University of British Columbia, Vancouver BC, Canada; 2 Department of Clinical Neuroscience, Imperial College London, London, UK

## Abstract

**Purpose:**

We recently demonstrated that molecular therapy using aminoglycosides can overcome the underlying genetic defect in two zebrafish models of ocular coloboma and showed abnormal cell death to be a key feature associated with the optic fissure closure defects. In further studies to identify molecular therapies for this common congenital malformation, we now examine the effects of anti-apoptotic compounds in zebrafish models of ocular coloboma in vivo.

**Methods:**

Two ocular coloboma zebrafish lines (*pax2.1*/*noi^tu29a^* and *lamb1*/*gup^m189^*) were exposed to diferuloylmethane (curcumin) or benzyloxycarbonyl-Val-Ala-Asp(Ome)-fluoromethylketone (zVAD-fmk; a pan-caspase inhibitor) for up to 8 days post-fertilization. The effects of these compounds were assessed by morphology, histology, terminal deoxynucleotidyl transferase dUTP nick end labeling (TUNEL) staining and western blot analysis.

**Results:**

The size of the coloboma in *gup* zebrafish mutants treated with diferuloylmethane was greatly reduced. In treated mutants a reduction in TUNEL staining and a 67% decrease in activated caspase-3 protein were observed. The release of cytochrome c from the mitochondria into the cytosol was reduced fourfold by in vivo diferuloylmethane treatment, suggesting that the drug was acting to inhibit the intrinsic apoptotic pathway. Inhibition of caspases directly with zVAD-fmk also resulted in a similar reduction in coloboma phenotype. Treatment with either diferuloylmethane or zVAD-fmk resulted in a statistically significant 1.4 fold increase in length of survival of these mutant zebrafish (p<0.001), which normally succumb to the lethal genetic mutation. In contrast, the coloboma phenotype in *noi* zebrafish mutants did not respond to either diferuloylmethane or zVAD-fmk exposure, even though inhibition of apoptotic cell death was observed by a reduction in TUNEL staining.

**Conclusions:**

The differential sensitivity to anti-apoptotic agents in *lamb1*-deficient and *pax2.1*-deficient zebrafish models, suggests that apoptotic cell death is not a final common pathway in all ocular coloboma genotypes. When considering anti-cell death therapies for ocular colobomatous defects attention should be paid to the genotype under investigation.

## Introduction

During early eye development a critical morphogenetic event is the formation and closure of the optic fissure to allow, for example, ingression of the hyaloid vasculature to supply the developing retina [1]. This process requires precisely coordinated sculpting and folding of epithelial tissue, so that the edges of the optic fissure can align, converge and fuse. The most common abnormality of this process, failure of this optic fissure to close, leads to the congenital malformation known as ocular coloboma, with an incidence of 0.5–7 per 10,000 births [2]. As an example of failure of developing tissue to fuse, ocular coloboma can be grouped along with other developmental anomalies such as spina bifida and cleft lip/palate [3-5]. Potentially affecting the retina, choroid and optic nerve as well as the iris, it has been reported in up to 11.2% of blind children worldwide [6] making this untreatable developmental abnormality an important area to study.

The first step in delineating the signaling pathways that lead to proper folding, apposition and fusion of developing eye tissue is to identify molecular events causing optic fissure closure defects [7-13]. Although a large number of genetic defects have been associated with ocular coloboma [14], the molecular and cellular mechanisms underlying this are poorly understood. Data from several sources has however emerged to suggest that apoptotic cell death pathways are abnormally activated in cases of coloboma [15,16]. These data suggest that modulation of apoptotic cell death could be a viable strategy for inhibiting coloboma formation independent of the underlying cause [17-19].

We had previously shown that the zebrafish model system is particularly suited to drug testing in vivo. In two ocular coloboma models we showed that translational bypass of the premature stop codon mutation in each mutant using aminoglycosides was able to rescue the colobomatous defect and concomitantly inhibit cell death in the eye [16]. In this study we proposed to identify other pharmaceutical approaches to modifying molecular pathways triggered by coloboma mutations.

Diferuloylmethane (commonly known as curcumin) is a natural polyphenol component found in the rhizome of the *Curcuma longa* plant. Extensive studies have revealed diverse pharmacological effects induced by diferuloylmethane reported both in vitro and in vivo, including anti-inflammatory [20], antioxidant [21], anti-tumor [22], and neuroprotective (anti-cell death) actions [23-26]. zVAD-fmk is a broad spectrum caspase inhibitor that is commonly used to investigate cell death signaling in vitro, and several studies have shown that it has beneficial anti-cell death effect in vivo. For example, it delays disease onset and extends the survival in the amyotrophic lateral sclerosis transgenic mouse model [27], it rescues renal hypoplasia in paired box 2 (*Pax2*)-deficient mice [28] and promotes cochlear hair cell survival in the *Pou4f3* mouse model of deafness [29]. In this study we tested the ability of diferuloylmethane and benzyloxycarbonyl-Val-Ala-Asp(Ome)-fluoromethylketone (zVAD-fmk) to attenuate the congenital coloboma phenotype in zebrafish models of ocular coloboma.

## Methods

### Zebrafish strains and maintenance

Zebrafish strains (wildtype AB [30], *noi^tu29a^* [31] and *gup^m189^* [32]) were maintained and staged according to morphological criteria using established protocols [33,34]. The *noi* strain has a nonsense mutation in the *pax2.1* gene with a mild coloboma phenotype, whereas the *gup* strain has a nonsense mutation in the laminin beta 1 (*lamb1*) gene with a severe coloboma phenotype. Research was carried in accordance with the principles and guidelines of The Animals (Scientific Procedures) Act 1986, UK, the Canadian Council of Animal Care and the ARVO statement for the use of animals in vision research. Embryos were raised at 28.5 °C on a 14 h light/10 h dark cycle in 100 mm^2^ Petri dishes containing aquaria water. To aid image analysis, 0.2 mM phenylthiourea (PTU; Sigma-Aldrich, Oakville, ON, Canada) was added to the embryos at 10 h post-fertilization (hpf) to inhibit pigment formation.

### Anti-apoptosis treatment

All chemicals were obtained from Sigma-Aldrich (Oakville, ON, Canada). Stock solutions of zVAD-fmk and zFA-fmk (negative control for zVAD-fmk) in dimethyl sulfoxide (DMSO) and diferuloylmethane in PBS (137 mM NaCl, 2.7 mM KCl, 4.3 mM Na_2_HPO_4_, and 1.47 mM KH_2_PO_4_, pH of 7.4) were added to aquaria water. Initial dose–response experiments in zebrafish embryos were performed to determine non-toxic dosages. Mutant embryos were dechorionated at 10 hpf and treated with diferuloylmethane, zVAD-fmk, zFA-fmk or kept in control (drug-free) aquaria water until either 6 or 8 days post-fertilization (dpf). An equivalent volume of DMSO was added to control cultures where necessary. For each treatment 30 embryos were used and three independent experiments were performed. Quantitative data are expressed as mean±SEM. Statistical analysis of the data by pair-wise comparisons between the control, untreated mutants and each treatment group was performed using the Mann–Whitney test. A p value <0.05 was considered significant.

### Retinal histology and morphological studies

Embryos were fixed in 4% paraformaldehyde (PFA) overnight at 4 °C before histological or terminal deoxynucleotidyl transferase dUTP nick end labeling (TUNEL) analysis analysis. After fixation in 4% PFA, embryos were washed 3 times in PBS and dehydrated through a graded ethanol series (50%, 70%, 90%, and 3 times in 100%). Embryos were transferred into cedar wood oil and then embedded in paraffin wax. Microtome sections of 5 µm thickness were cut, mounted on Superfrost Plus slides (Thermo Scientific, Ottawa, ON, Canada) and counterstained with hematoxylin and eosin. Images were captured with an EM-CCD camera (Hamamatsu Photonics Ltd, Welwyn Garden City, UK) using a widefield Axiovert microscope (Carl Zeiss Inc, New York, NY). Whole-mount morphological images were taken with a DFC300 FX camera (Leica Microsystems Ltd., Milton Keynes, UK) mounted on a Z16F stereomicroscope (Leica Microsystems Ltd.).

### TUNEL assay

PTU-treated embryos at 6 dpf from each treatment group were fixed in 4% PFA and embedded in wax as described above. Retinal sections were dewaxed by washing twice in Histoclear (National Diagnostics, Hessle, UK), followed by 2 washes in 100% ethanol and once in 70% ethanol, before rinsing in deionised H_2_O. For wholemount TUNEL assays, embryos were dehydrated through a graded methanol series (25%, 50%, 75%, and twice in 100%) and stored in 100% methanol at −20 °C. After rehydration, both sections and wholemount embryos were digested with proteinase K (10 μg/ml) for 15 min and 1 h, respectively. Embryos were refixed with 4% PFA for 20 min at room temperature (RT), followed by several washed in PBS. The ApopTag^®^ Peroxidase In Situ Apoptosis Detection Kit (Millipore, Bedford, MA) was used to detect levels of apoptotic cell death, following the manufacturer’s instructions.

### Western blot analysis

Specific primary antibodies were obtained from commercial sources and used as follows: 1:200 cleaved caspase-3 rabbit polyclonal and 1:2,000 glyceraldehyde 3-phosphate dehydrogenase (GAPDH) mouse monoclonal (Abcam, Cambridge, MA); 1:500 cytochrome c rabbit polyclonal (Santa Cruz Biotech, Santa Cruz, CA); 1:4,000 β-actin mouse monoclonal (Sigma-Aldrich, Oakville, ON, Canada); 1:1,000 cox IV mouse monoclonal (Acris Antibodies, San Diego, CA).

Briefly, 25 wildtype and mutant embryos at 6 dpf were snap frozen in liquid nitrogen and homogenized by sonication in lysis buffer (10 mM Tris pH 7.5, 10 mM NaCl, 1% SDS, 1× Protease Inhibitor Cocktail (Roche, Indianapolis, IN). Insoluble material was removed by a 10 min centrifugation (25,000× g). For subcellular fractionation, frozen embryos were homogenized in hypotonic buffer (50 mM HEPES 7.5, 10 mM NaCl, 1 mM DTT, 1× Protease Inhibitor Cocktail) by sonication. Nuclei and unbroken cells were pelleted by centrifugation at 800× g for 10 min. The supernatant was collected and centrifuged at 100,000× g for 1 h at 4 °C using a Beckman Optima Ultracentrifuge (Beckman Coulter, Mississauga, ON, Canada) to obtain cytosolic fractions (supernatant) and mitochondrial fractions (pellet). Protein concentration was determined by the DC protein assay (Bio-Rad, Mississauga, ON, Canada).

Equal amounts of protein (40 μg) were separated on a 12% SDS-polyacrylamide gel and transferred to Immobilon-FL membrane (Millipore, Bedford, MA). The membrane was incubated in 5% non-fat milk powder in PBS/0.1% Tween-20 (PBST) for 2 h at room temperature and incubated overnight at 4 °C, simultaneously with two primary antibodies raised in different hosts. Following 3 washes in PBST, the membrane was incubated in the dark for 1 h with two Li-COR secondary antibodies simultaneously (IRDye 680LT goat anti-rabbit; IRDye 800CW goat anti-mouse; Mandel Scientific, Guelph, ON, Canada). After the membrane was washed 3 times in PBST in the dark, protein bands were visualized using a Li-COR Odyssey detector. The Li-COR software and Image J were used to quantify band intensity.

## Results

### Therapeutic dose for diferuloylmethane and zVAD-fmk in zebrafish

**Figure 1:**
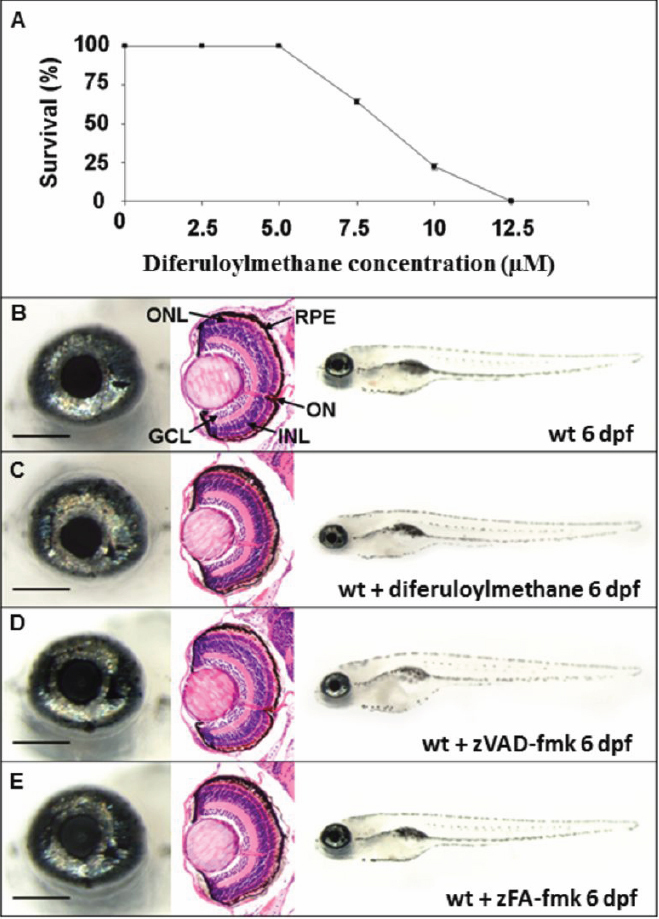
Effect of drug treatment on wildtype zebrafish. **A**: Wildtype embryos were dosed continuously from 10 hpf with diferuloylmethane. Percentage survival of embryos at 6 dpf was determined for each treatment group (30 embryos/group), n=3, mean±SEM. Error bars smaller than the symbol are not visible. **B-E**: Left panels, wholemount eye; center panels, coronal retinal section; right panels, wholemount larvae. **B**: Wildtype phenotype untreated at 6 dpf. **C**: Wildtype phenotype at 6 dpf treated with 5 µM diferuloylmethane from 10 hpf. **D**: Wildtype phenotype at 6 dpf following 300 µM zVAD-fmk treatment. **E**: Wildtype phenotype at 6 dpf following 300 µM zFA-fmk treatment. Scale bar left panel=200 µm. GCL, ganglion cell layer; INL, inner nuclear layer; ONL, outer nuclear layer; RPE, retinal pigment epithelium, ON, optic nerve.

To determine the highest dose of drug that could be tolerated, we performed survival experiments. The survival rates of wildtype embryos dosed continuously from 10 hpf (before optic fissure morphogenesis) with increasing concentrations of diferuloylmethane from 1 μM to 1 mM was measured at 6 dpf (Figure 1A), and behavioral and gross morphological defects were noted in surviving larvae. Embryos treated with 5 μM diferuloylmethane displayed normal morphology and behavior with 100% survival rates at 6 dpf, and retinal sections had a normal histological appearance at 6 dpf (Figure 1B,C). Doses of >5 μM yielded toxic side effects including pericardial edema, bent tails, spinal curvature, shorter body length, and abnormal swimming behavior. Doses of ≥12.5 μM were lethal in 100% of embryos. These results were consistent with previous studies examining the effects of diferuloylmethane on zebrafish development [35]. Hence, a 5 μM concentration was chosen as the optimum therapeutic dose for in vivo testing in zebrafish mutants. Similar dose response curves were performed for zVAD-fmk and zFA-fmk and the optimum dose of 300 μM for both compounds was determined to have no toxic side effects (Figure 1D,E).

### Effect of diferuloylmethane and zVAD-fmk on coloboma mutant morphology

**Figure 2:**
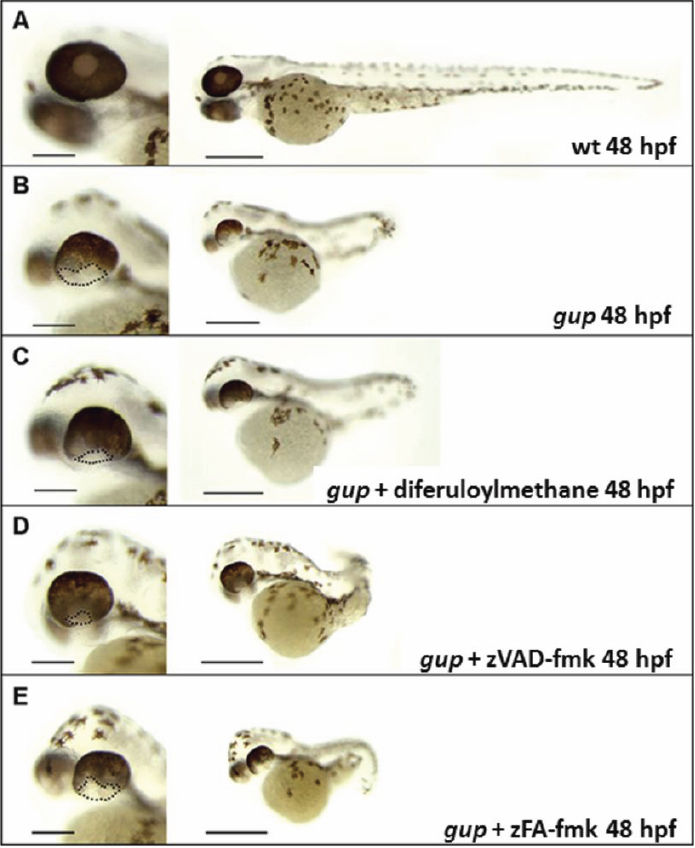
Representative morphology of *gup* mutants at 48 hpf following drug treatment. Left panel, enlarged image of wholemount eye, scale bar=200 μm. Right panel, whole embryo morphology, scale bar=500 μm. **A**: Wildtype control, complete closure of optic fissure. **B**: Untreated *gup* mutant, displaying large open optic fissure at ventral aspect of eye. **C**: *gup* mutant treated with 5 µM diferuloylmethane, displaying open optic fissure which is smaller in size compared to untreated mutants. **D**: *gup* mutant treated with 300 µM zVAD-fmk, displaying a smaller open optic fissure than untreated mutants. **E**: *gup* mutant treated with 300 µM zFA-fmk, displaying a large open optic fissure as in untreated mutants. Optic fissure closure defect delineated by black dotted line.

**Figure 3:**
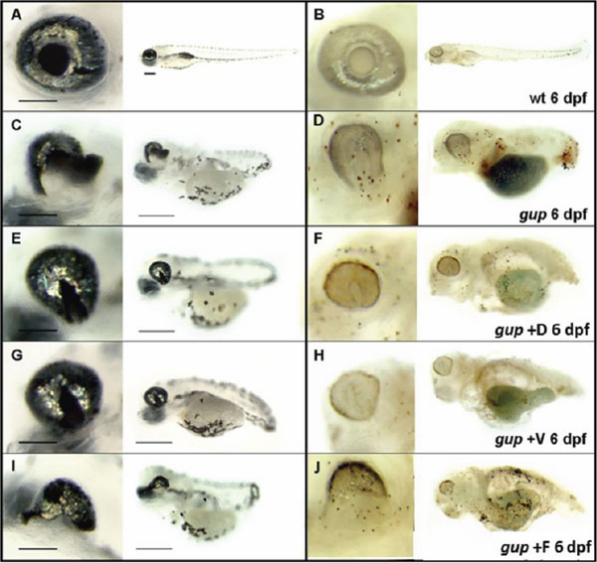
Representative images of drug treatment in *gup* mutants at 6 dpf. **A**, **C**, **E**, **G**, **I**: Enlarged wholemount of the eye, scale=200 µm, and wholemount larvae, scale=500 µm. **B**, **D**, **F**, **H**, **J**: corresponding TUNEL stained wholemount of PTU-treated eye and larvae. **A**, **B**: Wildtype zebrafish showing normal morphology and minimal apoptosis in the eye and wholemount. **C**, **D**: Untreated *gup* mutants displaying large coloboma in ventral aspect of the eye, TUNEL-positive labeled tissue at the site of the unfused optic fissure and throughout the whole fish. **E**, **F**: *gup* mutants treated with 5 µM diferuloylmethane (+D) showing small colobomatous defect, minimal TUNEL-positive staining in the eye, and reduced levels in the whole larvae compared to untreated mutants. **G**, **H**: *gup* mutants treated with 300 µM zVAD-fmk (+V) showing small colobomatous defect, minimal TUNEL-positive staining in the eye, and reduced levels in the whole larvae compared to untreated mutants. **I**, **J**: *gup* mutants treated with 300 µM zFA-fmk (+F) showing large colobomatous defect and TUNEL-positive staining at the site of the unfused fissure.

Mutant *gup* embryos were dosed with 5 μM diferuloylmethane or 300 μM zVAD-fmk at 10 hpf and incubated up to 6 dpf. At 48 hpf, treated *gup* mutants displayed a smaller optic fissure closure defect with both drugs (compare Figure 2A,B with Figure 2C,D), but with similar systemic features compared to untreated mutants. In contrast, the zFA-fmk control drug at a concentration of 300 µM had no effect of the size of the coloboma (Figure 2E). By 6 dpf, the size of the optic fissure was greatly reduced with both drugs revealing a persistent milder coloboma phenotype (compare Figure 3A,C with Figure 3E,G) whereas zFA-fmk had no effect on the coloboma phenotype (Figure 3I). To determine whether this effect of drug treatment was through a reduction of cell death, we performed TUNEL-staining (Figure 3B,D,F,H,J). In whole-mount analysis there was no TUNEL-positive staining at the position of the optic fissure in treated *gup* mutants compared to untreated mutants, suggesting that diferuloylmethane and zVAD-fmk had caused a reduction in apoptotic cell death (compare Figure 3D with Figure 3F,H).

**Figure 4:**
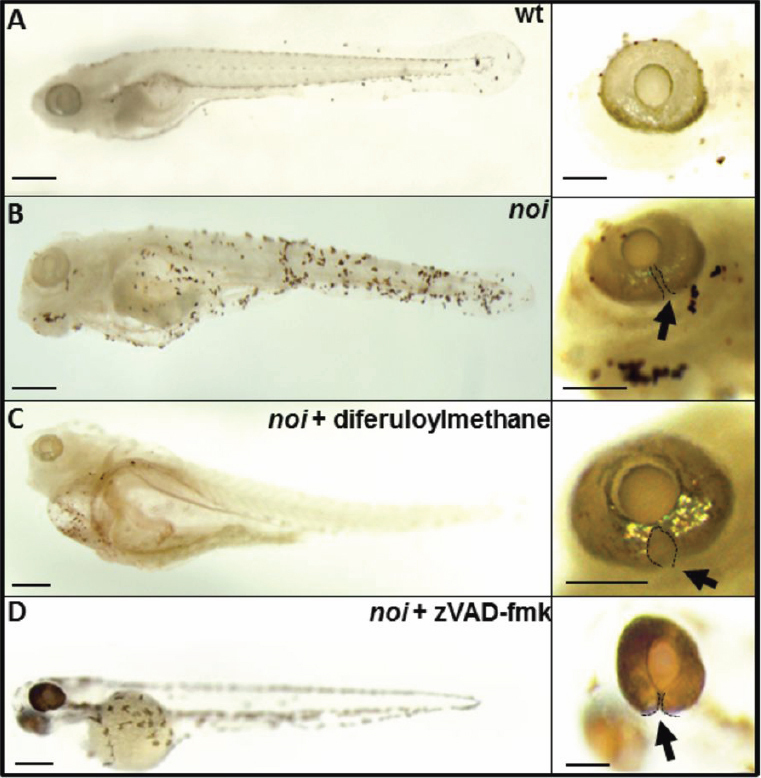
Representative images of the effect of diferuloylmethane on TUNEL-labeling in *noi* mutant larvae at 6 dpf. **A**: TUNEL-labeling in wholemount larvae (left) and wholemount eye (right) of wildtype zebrafish (wt). **B**: Extensive TUNEL labeling in larvae and eye of untreated *noi* mutant zebrafish. **C**: *noi* mutant larvae treated with 5 µM diferuloylmethane. **D**: *noi* larvae treated with 300 µM zVAD-fmk. Arrow points to optic fissure closure defect delineated by black dotted line. Scale bar whole eye=200 µm; scale bar larval fish=500 µm.

To determine whether diferuloylmethane or zVAD-fmk treatment would also be effective in other models of coloboma, these drugs were tested in the *noi* mutant model of coloboma from 10 hpf and incubated up to 6 dpf. When *noi* mutant fish were treated with 5 µM diferuloylmethane the amount of TUNEL-labeled cells present in wholemount fish was greatly reduced throughout the whole fish and the eye (compare Figure 4B with Figure 4C). Conversely however, the size of the coloboma appeared enlarged rather than reduced. Similarly, although 300 µM zVAD-fmk reduced TUNEL staining in the fish, it had no discernible effect on the coloboma defect (Figure 4D).

**Figure 5:**
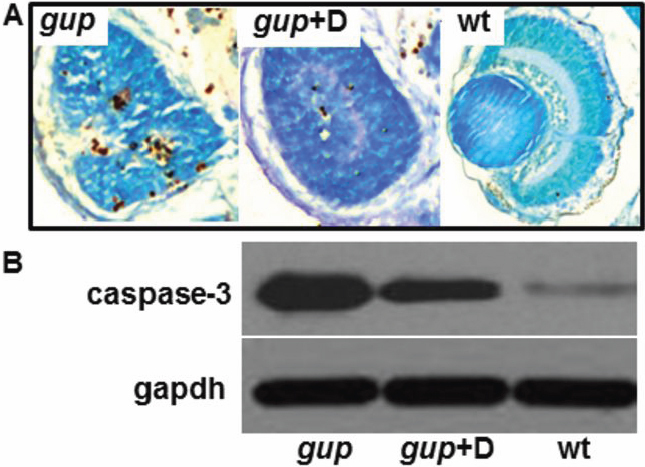
Effect of diferuloylmethane on cell death in *gup* mutant larvae. **A**: Comparison of TUNEL-positive cells in coronal cryosections of the eye from mutant *gup* embryos (left), mutant *gup* embryos treated with 5 µM diferuloylmethane (center) and wildtype embryos (right) at 6 dpf. Histological sections counterstained with methyl green. **B**: Representative western blot of lysates from untreated mutant larvae (*gup),* mutant larvae treated with 5 µM diferuloylmethane (*gup*+D) and wildtype larvae (wt). Upper lanes, correspond to cleaved caspase-3; lower lanes, corresponding gapdh loading controls.

To further explore this beneficial reduction in coloboma size seen with diferuloylmethane in *gup* zebrafish, we used TUNEL-staining to examine cell death in whole-eye sections at 6 dpf. Diferuloylmethane treatment again confirmed far fewer TUNEL-positive cells throughout the eye of mutant zebrafish compared to untreated zebrafish (Figure 5A). This experiment however showed that although the size of optic fissure was reduced, there was no formation of a lens with diferuloylmethane treatment.

### Effect of diferuloylmethane on caspase activity and mitochondrial cytochrome c release in *gup* zebrafish

**Figure 6:**
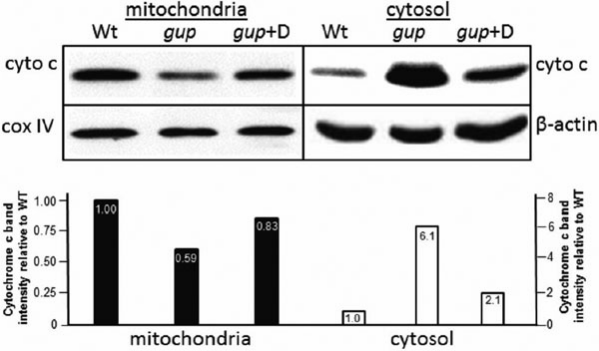
Representative western blots showing cytochrome c levels in *gup* mutants treated with diferuloylmethane. Cytochrome c (cyto c) levels were determined in embryos treated for 6 days. Mitochondrial and cytosolic fractions were normalized with cox IV or β-actin, respectively. Quantitation of western blot of cytochrome c localization in larvae is shown directly beneath western blots. Wildtype levels in each fraction are set to 1.0. Wt, wildtype; *gup,* untreated mutant larvae, *gup*+D, mutant plus diferuloylmethane.

To determine if the reduction in TUNEL-positive labeling caused by diferuloylmethane is a caspase-dependent process, protein extracts were obtained from 6 dpf embryos and tested for activated caspase-3 activity by western blot. Zebrafish lysates from *gup* mutant fish displayed high levels of cleaved caspase-3 activity (maximally 100%) compared to wildtype embryos (2.7%; Figure 5B). However, mutant embryos treated with diferuloylmethane had a 67% reduction in the level of cleaved caspase-3 protein. We therefore tested whether this reduction in caspase-3 activity was associated with inhibition of the intrinsic apoptotic pathway, by assessing whether cytochrome c was released from the mitochondria into the cytosol [36]. In wildtype zebrafish at 6 dpf, cytochrome c levels were highest in the mitochondrial fraction and were only detected at low levels in the corresponding cytosolic fraction (Figure 6). In untreated mutants at 6 dpf, cytochrome c was elevated sixfold in the cytosol, with a corresponding reduction in the mitochondrial fraction. However, when *gup* mutants were treated with diferuloylmethane from 10 hpf to 6 dpf, the release of cytochrome c into the cytosol was greatly reduced to just twofold over wildtype levels.

### Effect of diferuloylmethane and zVAD-fmk on survival

**Figure 7:**
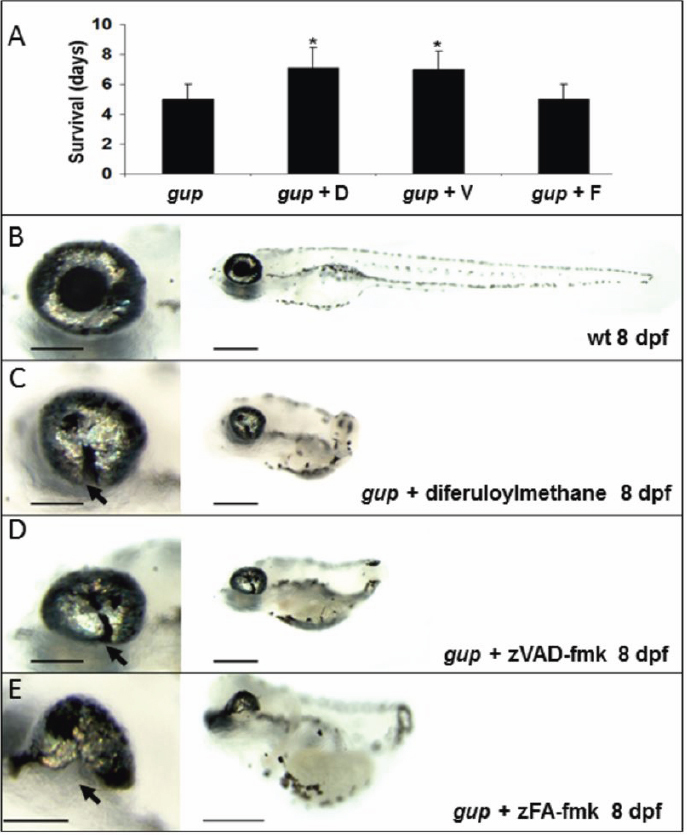
Effect of caspase inhibition on survival and optic fissure closure. **A**: Mean survival of *gup* mutant embryos with no treatment or with either diferuloylmethane, zVAD-fmk or control inhibitor zFA-fmk. n*=*30 for each group, mean±SEM (*p<0.001). **B**: Wildtype (wt) larval phenotype at 8 dpf. **C**: Phenotype of *gup* mutants treated with 5 µM diferuloylmethane. **D**: Phenotype of *gup* mutant embryos treated with 300 µM zVAD-fmk. **E**: Phenotype of *gup* mutants treated with 300 μM zFA-fmk. Arrows indicate the position of the coloboma. Size bar in left panels=200 μm; size bar in right panels=500 μm.

Since *gup* mutants showed extensive TUNEL labeling throughout the larva, which was reduced by the presence of diferuloylmethane, we speculated whether the mutant larva would survive past its usual life-span of only 5 days (the *lamb1* mutation is embryonic lethal). Mutant embryo survival was therefore tested in the presence of either: diferuloylmethane, zVAD-fmk or zFA-fmk. The presence of diferuloylmethane from 10 dpf increased the embryo life-span from 5.0±1.0 days to 7.1±1.4 days in treated embryos (Figure 7A), representing a statistically significant (p<0.001)1.4 fold increase in survival. Similarly, zVAD-fmk also increased embryo survival from 5.0±1.0 days to 7.0±1.5 days (p<0.001). No effect on embryo survival was observed with zFA-fmk. Furthermore, the size of the coloboma was even smaller in mutants treated with curcumin or zVAD-fmk at 8 dpf (Figure 7C,D) compared to 6dpf (Figure 3E,G).

## Discussion

This study demonstrates that an anti-cell death strategy (using diferuloylmethane or zVAD-fmk) not only substantially ameliorates the ocular coloboma phenotype in the *gup* (*lamb1*) zebrafish but also increase embryo survival rate. Our results also suggest that this reduction in disease severity in the *gup* model occurs through inhibition of the intrinsic (mitochondrial) apoptotic pathway via a caspase-3 dependent mechanism. Persistence of an albeit smaller colobomatous defect, and absence of the lens with diferuloylmethane however suggests that this benefit is partial. Contrastingly, in a second coloboma model where *pax2.1* is deficient (*noi* line), apoptotic cell death in the eye was again reduced by diferuloylmethane and zVAD-fmk, but we could detect no improvement in phenotype, suggesting that caspase-dependent cell death is not critical in the pathogenesis of ocular disease in *noi* zebrafish.

Of the many different mechanisms at play during normal neurodevelopment, cell death, specifically apoptotic cell death has emerged as an important, late event [37]. During eye development, we have shown that abnormal apoptotic cell death can be triggered during pathological states [16]. Although the in vivo effects of diferuloylmethane are thought to be diverse [20-22], our TUNEL and western blot studies suggest that, in the *gup* zebrafish, diferuloylmethane’s effects are due to an inhibition of cell death through an attenuation of the intrinsic, mitochondrial apoptotic pathway. This is in keeping with other studies, both in vitro [23,25] and in vivo [24,26,38]. Our data therefore supports the conclusion that mitochondrial cytochrome c release, caspase activation and apoptotic cell death are critical elements of ocular coloboma pathogenesis in the *gup* zebrafish and this can be inhibited by diferuloylmethane treatment. Previous safety and bioavailability studies [39-41] suggest that diferuloylmethane is a viable option for further pre-clinical studies in mammalian model systems. However, since ocular coloboma is a congenital anomaly, in utero safety and bioavailability studies would be needed before undertaking clinical trials of diferuloylmethane or one of its more efficacious analogs.

Contrastingly, our studies in the *noi* zebrafish suggest that such a strategy may not be universally applicable in all cases of ocular coloboma. Although results suggest that apoptotic cell death is triggered and can be inhibited in the *noi* zebrafish, this does not prevent the evolution of a colobomatous eye. A comparable situation is seen in some retinal dystrophy mutants where photoreceptor cell death is a common if not universal feature [17,19]. Not all mutants however respond to inhibitors of apoptotic cell death [42-44]. This leads us to speculate that other cell death mechanisms may be at play in the *noi* coloboma model.

Uncharacterized mechanisms other than cell death may have more critical influences on coloboma pathogenesis in this *noi* zebrafish. It is however possible that other cell death mechanisms in addition to caspase-dependent cell death, are at play and are more important in the pathogenesis of this model. Inhibitors of calpain [45] and poly (ADP-ribose) polymerase (PARP) [46], key elements in caspase-independent cell death, have for example been shown to be effective in some retinal degeneration models. In addition, it has been shown that in the absence of caspase-dependent cell death, autophagy cell survival mechanisms can be activated to stimulate cell death [47,48]. Autophagic cell death has been shown to be important in normal embryologic development [49] and play a role in neural tube defects, an epithelial fusion defect similar to ocular coloboma [50]. Inhibitors of autophagy have thus been effective in neuronal cell survival [51] and hepatic fibrosis [52]. Necroptosis, regulated necrosis without inflammation, has also recently been highlighted as an alternative cell death pathway [53,54]. A role for necroptosis has been established in eye disease [55] and neurodegeneration [56], but a convincing role in developmental disease has yet to be shown [57]. The relevance of these alternate cell death pathways to the pathogenesis of ocular coloboma awaits further study, however it is likely that cross-talk between many different cell death pathways [58] determines final cell termination events.

In conclusion, this study highlights caspase-3 dependent cell death as a critical contributor in some, but not all inherited forms of optic fissure closure defects. As more becomes known about the different cell death pathways and the associated genotype-phenotype correlations, then more specific therapies for different cell death mechanisms can be formulated.
